# Effect of Methyl Jasmonate on the Growth, Antioxidant Potential, and Phenolic Compound Synthesis of *Arnica montana* L. In Vitro Shoots

**DOI:** 10.3390/biology15120909

**Published:** 2026-06-10

**Authors:** Mirena Chakarova, Kamelia Miladinova-Georgieva, Maria Geneva, Mariana Sichanova, Antoaneta Trendafilova, Viktoria Ivanova, Magdalena Sozoniuk, Lyudmila Dimitrova, Margarita Dimitrova, Milena Nikolova, Maria Petrova

**Affiliations:** 1 Institute of Plant Physiology and Genetics, Bulgarian Academy of Sciences, Acad. G. Bonchev Street, Bldg. 21, 1113 Sofia, Bulgaria; mchakarova@bio21.bas.bg (M.C.); kameca@abv.bg (K.M.-G.); boykova2@yahoo.com (M.G.); m.sichanova@abv.bg (M.S.); dim.lyudmila@gmail.com (L.D.); mstoyadinova@abv.bg (M.D.); 2 Institute of Organic Chemistry with Centre of Phytochemistry, Bulgarian Academy of Sciences, Acad. G. Bonchev Street, Bldg. 9, 1113 Sofia, Bulgaria; antoaneta.trendafilova@orgchm.bas.bg (A.T.); viktoria.genova@orgchm.bas.bg (V.I.); 3 Centre of Competence “Sustainable Utilization of Bio-Resources and Waste of Medicinal and Aromatic Plants for Innovative Bioactive Products” (BIORESOURCES BG), Acad. G. Bonchev Street, Bldg. 9, 1113 Sofia, Bulgaria; 4 Institute of Plant Genetics, Breeding and Biotechnology, University of Life Sciences in Lublin, 20-950 Lublin, Poland; magdalena.sozoniuk@up.lublin.pl; 5 Institute of Biodiversity and Ecosystem Research, Bulgarian Academy of Sciences, Acad. G. Bonchev Street, Bldg. 23, 1113 Sofia, Bulgaria; mtihomirova@gmail.com

**Keywords:** in vitro shoots, elicitation, total phenolic content, total flavonoid content, caffeoylquinic acids, MeJA

## Abstract

*Arnica montana* L. is a valuable medicinal plant with strong anti-inflammatory properties attributed to its high levels of antioxidants and specific metabolites. The species is endemic to Europe, and its natural populations are threatened by habitat disturbance and poor management. In vitro plant cultures are an alternative method for rapid plant multiplication and the controlled synthesis of biologically active substances. Elicitation is a common strategy for inducing physiological changes and defense responses in plants, thereby increasing their antioxidant potential and the synthesis of specialized metabolites. Methyl jasmonate, a plant growth regulator involved in the modulation of plant growth and development at morphological and molecular levels, has gained increasing attention as an elicitor. In the present study, three concentrations of MeJA (50, 100, and 200 μM) were applied for 3 and 7 days to evaluate their effects on growth, antioxidant capacity, and accumulation of caffeoylquinic acids in in vitro shoots of arnica. Seven-day MeJA treatment reduced growth parameters and was accompanied by increased activities of antioxidant enzymes SOD and APX, as well as elevated levels of water-soluble antioxidants, which correlated with the enhanced accumulation of caffeoylquinic acids, total phenolics, and flavonoids. The results demonstrate that MeJA affected shoot growth and antioxidant potential of *A. montana*, with concentration and treatment duration playing a critical role in response magnitude.

## Simple Summary

*Arnica montana* has been used for centuries in medicine for a variety of therapeutic purposes. The extract of the plant possesses diverse biological activities, such as anti-inflammatory, antifungal, antiviral, antitumor, tissue-regenerative, etc. The increasing market demand for arnica-containing products requires the development of an effective method for the rapid propagation of the plant, as well as the production of biomass and important pharmaceutical metabolites. Methyl jasmonate (MeJA) is an effective tool for enhancing the production of secondary metabolites in in vitro cultures. It is very useful in the synthesis of pharmaceutically significant chemicals because it mimics stress signals and activates complex metabolic pathways. For the first time, the effect of the exogenous application of MeJA on micropropagation, antioxidant capacity, and caffeoylquinic acid accumulation in arnica in vitro plantlets was evaluated. The results showed that 7-day treatment with MeJA (50, 100 and 200 μM) inhibited plant growth, modulated the activity of antioxidant enzymes, and upregulated the content of caffeoylquinic acids, total phenolics, and flavonoids. This work provides a reliable in vitro culture system for the accumulation of *A. montana* phenolic compounds.

## 1. Introduction

*Arnica montana* L. is a perennial herbaceous plant that belongs to the family Asteraceae and has a long history of medicinal use dating from ancient times. The bioactive compounds identified in *A. montana* possess a range of biological properties—antibacterial, antifungal, antioxidant, anti-inflammatory, anti-sclerotic, anti-HIV, anticancer, etc. [1,2]. Due to intensive harvesting, some of its natural populations have disappeared, while others have declined in size and abundance. For this reason, the species is included in the Red Lists of several European countries and is subject to national conservation assessments and measures [3]. In vitro plant cultures represent a sustainable strategy for cultivating medicinal plants and obtaining bioactive compounds, helping to mitigate the overexploitation of natural resources [4,5]. The production of secondary metabolites from in vitro cultures has several advantages over their extraction from traditionally cultivated plants [6]. Among these are the absence of seasonal restrictions, shorter production cycles, and the potential for predictable, reliable, and market-specific production. There are various protocols for the clonal propagation of *A. montana* based on the supplementation of the nutrient media with cytokinins (zeatin, kinetin, 6-benzylaminopurine, and 2-isopentenyl adenine) and/or auxins (α-naphthaleneacetic acid, indole-3-acetic acid, and 2,4-dichlorophenoxyacetic acid) [7,8,9]. Elicitation is a relatively new, highly effective approach for increasing the synthesis of target biologically active compounds in vivo and in vitro by inducing signal transduction and activating plant defense mechanisms and different metabolic pathways [10,11]. Based on their origin, elicitors are classified into two categories: abiotic and biotic [12]. Abiotic elicitors encompass physical and chemical factors, including UV radiation, osmotic stress, drought, salinity, temperature stress, and heavy metals. They also include hormonal compounds, for example, intracellular signaling molecules like jasmonic acid, methyl jasmonate, salicylic acid, polyamines, abscisic acid, gibberellic acid, and gaseous molecules such as ethylene and nitric oxide [13]. Biotic elicitors are substances of biological origin, including polysaccharides and pathogen-derived components such as extracts from yeasts, bacteria, and fungi [14].

Methyl jasmonate (MeJA) is the methyl ester of jasmonic acid, frequently used as an elicitor. It stimulates the biosynthesis of secondary metabolites in plants by initiating signal transduction pathways, inducing the expression of key genes, and enhancing enzymatic activity [15,16]. Positive effects of MeJA on the synthesis of secondary metabolites have been documented in several plant species grown in vitro: silymarin and phenolic acids in callus and hairy root cultures of *Silybum marianum* L. [17]; stevioside and total phenolics in in vitro propagated *Stevia rebaudiana* Bert. [18]; chicoric acid in suspension culture of *Echinacea purpurea* L. [19]; artemisinin in hairy root culture of *Artemisia annua* L. [20]; glycyrrhizin in shoot culture of *Glycyrrhiza glabra* L. [21]; 3,5-dicaffeoylquinic acid in hairy root culture of *Cichorium intybus* L. [22], etc. In addition, JA signaling plays a role in the reprogramming of nitrogen uptake and metabolism [23].

The main bioactive compounds of *A. montana* are sesquiterpene lactones of the pseudoguaianolide type, specifically helenalin, 11α,13-dihydrohelenalin, and their esters. Other important groups of compounds identified include glucuronides, flavonoids (glycosides and aglycones), essential oils, thymol derivatives, inulin, carotenoids, and tannins [24]. The aerial parts of *A. montana* contain flavonoids and phenolic acids, particularly derivatives of caffeoylquinic acids (esters of caffeic acid and quinic acid) involved in the plant’s antioxidant defense and anti-inflammatory activity [25,26]. The caffeoylquinic acids (CQAs) have been repeatedly identified by LC–MS and HPLC studies as abundant constituents of *Arnica* species. They are often used as important markers in phytochemical profiling and quality control of herbal preparations [25,26,27,28]. Studies investigating the elicitation of *A. montana* in vitro shoots with jasmonic acid or MeJA have mainly focused on stimulating sesquiterpene lactone biosynthesis [29,30]. The limited production of sesquiterpene lactones following MeJA treatment has been demonstrated in our previous study [30]. This indicates that MeJA may not efficiently stimulate transcription factors directly associated with the sesquiterpene lactone biosynthetic pathway. Alternatively, the elicitation may redirect metabolic flux towards other secondary metabolic pathways, particularly the phenylpropanoid pathway, closely associated with the biosynthesis of phenolic compounds. Phenolics play a crucial role in plant defense responses and antioxidant activity, and their production is frequently enhanced under elicitor-induced stress conditions [31,32]. Therefore, investigating changes in total phenolic and flavonoid contents, as well as phenolic profiles following MeJA treatment, could provide valuable insights into the metabolic reprogramming occurring in response to elicitation. To the best of our knowledge, no studies have yet examined the effects of MeJA on phenolic compound levels and the antioxidant defense system in micropropagated *A. montana*.

Thus, the purpose of the current study was to investigate the effect of different concentrations and exposure times of MeJA on the growth, antioxidant potential, and caffeoylquinic acid synthesis in *A. montana* in vitro shoots.

## 2. Materials and Methods

### 2.1. Initiation of In Vitro Culture and Micropropagation of A. montana

Seeds of *A. montana* (collected from the experimental field of the Department of Industrial and Medicinal Plants at the University of Life Sciences in Lublin, Poland) were sterilized according to standard procedures using ethanol and commercial bleach, as previously described [33].

Explants were taken from in vitro germinated seeds and cultured in test tubes containing 6 mL Murashige and Skoog medium (macronutrients, micronutrients, and vitamins) [34], supplemented with 30 g/L sucrose, growth regulators: 6-benzylaminopurine (BAP) and indole-3-acetic acid (IAA), and 6 g/L agar. One explant was placed in each test tube. Plants were cultivated at a temperature of 22 °C, light intensity of 40 µmol m^−2^ s^−1^, and a photoperiod of 16 h light/8 h dark.

### 2.2. Treatment with MeJA

The stock solution of MeJA—1.03 g/mL (95% purity, Sigma-Aldrich, Schnelldorf, Germany) was dissolved in ethanol and subsequently diluted with distilled water. The MeJA solution was filtered through a 0.22 μm sterile filter (Minisart^®^, Sartorius, Göttingen, Germany) and then added aseptically to MS medium supplemented with 0.5 mg/L BAP at final concentrations of 50, 100, and 200 µM. The final amount of ethanol introduced into the culture medium was minimal and is unlikely to have substantially influenced the observed physiological and biochemical responses. Four-week-old shoot clumps (without dividing into separate shoots) grown on control MSB0.5 medium were transferred to MSB0.5 medium supplemented with the three previously described MeJA concentrations for periods of 3 and 7 days. Forty tubes containing 4-week shoot clumps were used per treatment. Control shoot clumps were transferred to MSB0.5 medium without MeJA. In vitro cultures were cultivated at a temperature of 22 °C, light intensity of 40 mol m^−2^ s^−1^, and a photoperiod of 16 h light/8 h dark. Shoots were harvested on the 3rd and 7th days of treatment. The average number of shoots per explant, the average plant height, and the average fresh weight were recorded. The experiments were carried out in triplicate. The pH of all media was adjusted to 5.7 before autoclaving.

### 2.3. Measurement of the Antioxidant Activity of Extracts from In Vitro Plants After Treatment with MeJA

Fresh plant material (0.5 g) was homogenized in 5 mL of 0.1 M phosphate buffer (pH 7.8) on ice. The buffer contained 2 mM EDTA, 10% glycerol, 2% polyvinylpyrrolidone (PVP), and 1 mM phenylmethylsulfonyl fluoride (PMSF). The resulting extract was centrifuged at 12,500 rpm for 30 min at 4 °C. The supernatant was used for protein quantification and enzymatic activity assays.

Protein content was determined using the Bradford method [35].

The activity of superoxide dismutase (SOD, EC 1.15.1.1) was determined based on the spectrophotometric measurement of the reduction of nitroblue tetrazolium (NBT) in the enzyme extract. The enzyme catalyzes the following reaction:

O_2_^−^ + O_2_^−^ + 2H^+^ → H_2_O_2_ + O_2_

The reaction mixture contained 50 mM potassium phosphate buffer (pH 7.8), 3.3 µM riboflavin, 10 mM methionine, 33 µM NBT, 0.66 mM EDTA, and the plant enzyme extract. The reaction mixtures were exposed to fluorescent light for 10 min, which photochemically induces the production of superoxide anion radicals through riboflavin irradiation in the presence of methionine. The radicals reduced the yellow NBT to a blue-colored formazan, which is insoluble. Inhibition of NBT reduction (decrease in absorbance) in the presence of the enzyme sample, compared to the control, was measured spectrophotometrically at 560 nm. One unit of specific SOD activity is defined as the amount of enzyme protein (mg) causing 50% inhibition of NBT photoreduction [36].

Catalase (CAT, EC 1.11.1.6) activity was measured using the method of Beers and Sizer [37], which is based on the enzyme’s ability to decompose hydrogen peroxide into water and oxygen. The reaction mixture contained 100 mM phosphate buffer (pH 7), 0.1 M H_2_O_2_, and 0.1 mL of enzyme extract. The activity was determined spectrophotometrically by the decrease in absorbance at 240 nm due to H_2_O_2_ decomposition over one minute, compared to a control sample (without plant extract).

Ascorbate peroxidase (APX, EC 1.11.1.11) activity was determined according to the method of Nakano and Asada [38]. The reaction mixture consisted of 0.05 mM ascorbic acid, 0.5 mM H_2_O_2_, 100 mM potassium phosphate buffer (pH 7.0), and 0.1 mL of enzyme extract. The decrease in absorbance at 290 nm was recorded over one minute. Enzyme activity is expressed per mg of protein.

Guaiacol peroxidase (GPX, EC 1.11.1.7) activity was measured following the method of Urbanek et al. [39]. The reaction mixture included 100 mM phosphate buffer (pH 7.0), 12.3 mM H_2_O_2_, 20 mM guaiacol, and 0.1 mL of enzyme extract. The increase in absorbance at 470 nm, resulting from the oxidation of guaiacol to tetraguaiacol over one minute, was monitored.

### 2.4. Determination of Non-Enzymatic Antioxidants (Phenolics and Flavonoids, Water- and Lipid-Soluble Antioxidants)

For the analysis of total phenolics and flavonoids, 0.3 g of dry plant material was placed in graduated test tubes with pre-heated 80% (*v*/*v*) methanol and incubated for 30 min in a water bath with continuous stirring. The sample was filtered, re-extracted, and filtered again, and the resulting residue was finally washed with 2 mL of methanol. The obtained supernatants were combined, brought to the same volume after cooling, and stored at low temperature.

Total phenolic content (TPC) was determined using the method of Pfeffer et al. [40]. The reaction mixture contained 0.1 mL of extract, 2.9 mL of distilled water, and 0.25 mL of Folin–Ciocalteu reagent. After 3 min, the reaction was stopped by adding 0.5 mL of 20% Na_2_CO_3_. After 1 h, the absorbance was measured at 725 nm wavelength against a blank sample containing 0.1 mL of 50% methanol. A calibration curve prepared with caffeic acid was used for the quantitative determination of total phenolics.

The method of Zhishen et al. [41] was used to determine the total flavonoid content (TFC). The reaction mixture included distilled water, the enzymatic extract (or an aliquot of catechin standard solutions), and 5% NaNO_2_. After 5 min, 10% AlCl_3_ was added, followed by 1 M NaOH after another 6 min. The solution was homogenized and left to stand for 30 min at room temperature. The method is based on the ability of flavonoids to form an aluminum complex with maximum absorbance at 510 nm. Absorbance was measured against a blank sample. Flavonoid content was determined using a standard curve prepared with catechin.

To extract water- and lipid-soluble antioxidant metabolites, 0.3 g of dry plant material were incubated in water and hexane, respectively, in a water bath with continuous stirring. The resulting extracts were filtered and brought to the same final volume. The reaction mixture contained 0.1 mL extract, 0.1 mL H_2_O_2_, 0.6 M H_2_SO_4_, 28 mM NaH_2_PO_4_, 4 mM (NH_4_)_6_Mo_7_O_24_·4H_2_O. The method is based on the reduction of Mo(VI) to Mo(V) and the formation of a green phosphate complex, which is dependent on the ascorbate and α-tocopherol equivalents in the sample. Absorbance was measured at 695 nm [42].

### 2.5. Total Antioxidant Activity (Ferric-Reducing Antioxidant Power—FRAP)

The same extracts used to isolate total phenols and flavonoids were additionally used for the FRAP assay. The ferric reducing antioxidant power assay (FRAP method) is based on the reduction of the ferric tripyridyltriazine (Fe(III)-TPTZ) complex to the ferrous tripyridyltriazine (Fe(II)-TPTZ) by antioxidants at low pH. The reaction mixture contains plant extract, FRAP reagent (prepared from acetate buffer, pH 3.6; a solution of 2,4,6-tripyridyl-s-triazine in 0.04 M HCl; and a solution of FeCl_3_·6H_2_O), and distilled water. The reaction takes place for 15 min at room temperature. A blue coloration develops, which is measured at 593 nm against a blank sample (containing methanol instead of extract) [43].

### 2.6. Qualitative and Quantitative Analyses of Caffeoylquinic Acids

#### 2.6.1. Preparation of the Extracts

One hundred milligrams of dried plant material were extracted with 2 mL of methanol at room temperature in an ultrasonic bath for 30 min. The obtained extract was centrifuged, filtered, and made up to 2 mL with methanol. One milliliter of the extract was passed through a Chromabond^®^ solid-phase extraction cartridge (C18ec, 500 mg, 3 mL, Marchery-Nagel, GMBH&Co., KG, Duren, Germany) to remove chlorophylls. Before analysis, the samples were filtered through a 0.22 μm syringe filter.

#### 2.6.2. High-Performance Thin-Layer Chromatography (HPTLC)

The qualitative HPTLC analysis was performed with precoated HPTLC glass plates (20 × 10 cm, Silicagel 60 F_254_, Merck, Germany) using a Camag HPTLC system (Camag, Switzerland). The mobile phase was toluene:ethyl acetate:formic acid:water (5:100:10:10, *v*/*v*/*v*/*v*). The application volume of the samples and standard solution was 5 µL. The standard solution was prepared from an equal amount of chlorogenic acid, 3,4-, 3,5-, 1,5- and 4,5-dicaffeoylquinic acids in methanol (0.2 mg/mL) purchased from PhytoLab GmbH & Co. KG, Germany. The chromatographic spots were visualized by UV light at 366 nm after dipping in NP reagent (5 mg/mL 2-aminoethyl diphenyl borate in ethyl acetate) and heating at 105 °C for 5 min.

#### 2.6.3. Quantitative Determination of Caffeoylquinic Acids by HPLC-PDA

The HPLC analysis was performed using a Shimadzu Nexera-i LC-2040C 3D Plus liquid chromatograph equipped with a photodiode array detector on an analytical Force C18 column (150 × 4.6 mm, 3 µm) at 30 °C. Elution was carried out in gradient mode using a mixture of 0.1% formic acid in water (A) and methanol (B). The chromatographic conditions and preparation of the calibration curves are described in detail by Ivanova et al. [44]. The concentration ranges, regression equations, correlation coefficients (R^2^), limit of detection (LOD) and limit of quantification (LOQ) of chlorogenic acid (5-CQA), 3,4-, 3,5-, 1,5- and 4,5-dicaffeoylquinic acids (DCQA) are given in Table 1. The detection was performed at 320 nm. The experiments were carried out in triplicate, and the results were expressed as mg/g DW (dry weight).

### 2.7. Statistical Analysis

The experiment was conducted in triplicate, representing three independent biological experiments performed under identical conditions. In each independent experiment, a total of 40 culture tubes containing explants were evaluated and distributed among all treatments, resulting in 120 culture tubes in total across all biological replicates.

For the biochemical analyses and metabolite assays, pooled plant material collected from the tubes within each independent replicate was used. Thus, one pooled sample was prepared for each of the three independent experiments, and the reported values represent the mean ± standard error calculated from these three biological replicates. Data were subjected to a one-way ANOVA analysis of variance for comparison of means, and significant differences were calculated according to the Fisher LSD test at the 5% level using a statistical software package (Statgraphics Plus, version 5.1 for Windows).

## 3. Results

### 3.1. Effect of MeJA on Shoot Growth and Development

Four-week-old shoot clumps were exposed to MeJA at concentrations of 50, 100, and 200 µM for 3 and 7 days. The number of shoots recorded after a 3-day treatment with MeJA averaged between 3.9 and 4.2 new shoots. There were no statistically significant differences compared to the control group, which had an average of 4.4 shoots per explant (Table 2). Plant height and fresh weight decreased in response to MeJA treatment.

**Figure 1:**
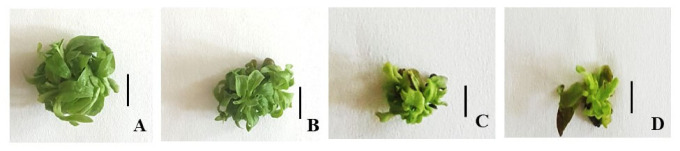
Shoot culture of *A. montana* L. after 7-day treatment with MeJA. (**A**) Control without MeJA, (**B**) 50 µM MeJA, (**C**) 100 µM MeJA, and (**D**) 200 µM MeJA. The scale bar represents: 1 cm.

Treatment with MeJA for 7 days resulted in a decrease in all of the examined morphometric parameters (number of shoots per explant, shoot height, fresh weight), with the lowest values recorded at the highest elicitor concentration (Table 2, Figure 1). Some of the shoots treated with 200 µM MeJA showed signs of necrosis and died.

### 3.2. Effect of MeJA on Antioxidant Enzyme Activity

**Figure 2:**
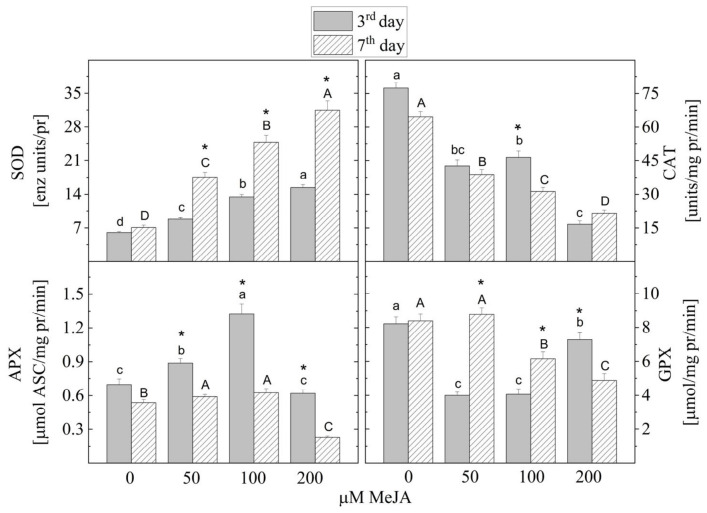
The activity of antioxidant enzymes superoxide dismutase (SOD), catalase (CAT), ascorbate peroxidase (APX), and guaiacol peroxidase (GPX) in *A. montana* plantlets elicited with MeJA applied at different concentrations (0, 50, 100, and 200 µM) on the 3rd and 7th day of the treatment. Values are means ± SE, *n* = 3; different letters indicate significant differences assessed by the Fisher LSD test (*p* ≤ 0.05) after performing ANOVA one-way analysis. We used the letter ‘a’ or “A” for the highest data value and descended to the next for lower data values. The statistical analysis of the 3rd day (lowercase) and 7th day (uppercase) was performed separately. The (*) indicates a significant difference between 3rd and 7th day in the same studied MeJA concentration.

The activity of the antioxidant enzyme SOD rose with increasing MeJA concentration in the nutrient medium and longer treatment duration (Figure 2). The maximum SOD activity was observed following treatment with the highest MeJA concentration (200 μM), with values on day 7 being four times higher than those in the control plants.

Catalase activity was higher in untreated control plants than in MeJA-treated plantlets on the 3rd and 7th day. When plants were treated with MeJA in all three tested concentrations, a decrease in the levels of CAT activities was observed, being more significant on the seventh day. The lowest CAT activity was reported at 200 μM MeJA on day 3 (Figure 2). Overall, CAT activity was higher after 3 days than after 7 days, except in plants treated with 200 μM MeJA. Treatment with 50 and 100 μM MeJA resulted in increased APX activity compared with the control, particularly after three days of treatment (Figure 2). The lowest APX activity was recorded on the 7th day of treatment with 200 μM MeJA. GPX activity decreased following MeJA treatment (Figure 2). On day 3, the highest GPX activity was observed in the control plants, followed by the plants treated with the highest MeJA concentration (200 μM). On day 7, GPX activity in plants treated with 50 μM MeJA remained close to the control, whereas higher elicitor concentrations led to a reduction in activity.

### 3.3. Effect of MeJA on the Content of Metabolites with Antioxidant Power (Total Phenols, Total Flavonoids, Water-Soluble Antioxidants WS-AOM, and Lipid-Soluble Antioxidants LS-AOM)

**Figure 3:**
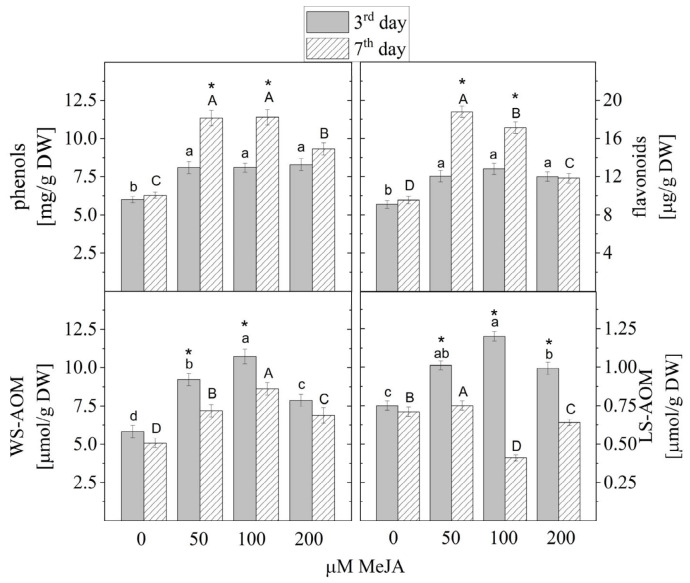
The content of metabolites with antioxidant power (total phenols, total flavonoids, water-soluble antioxidants WS-AOM, and lipid-soluble antioxidants LS-AOM) in *A. montana* plantlets elicited with MeJA applied at different concentrations (0, 50, 100, and 200 µM) on the 3rd and 7th day of the treatment. Values are means ± SE, *n* = 3; different letters indicate significant differences assessed by the Fisher LSD test (*p* ≤ 0.05) after performing ANOVA one-way analysis. We used the letter ‘a’ or “A” for the highest data value and descended to the next for lower data values. The statistical analysis of the 3rd day (lowercase) and 7th day (uppercase) was performed separately. The (*) indicates a significant difference between 3rd and 7th day in the same studied MeJA concentration.

Total phenolic content increased following treatment with MeJA, with the magnitude of the change depending on both the applied concentration and the duration of treatment (Figure 3). A rise in phenolic levels was observed as early as the third day of treatment compared with the untreated plants. However, no significant differences in phenolic levels were detected among *A. montana* plantlets treated for 3 days with different MeJA concentrations (50–200 µM) in the MS medium. A seven-day treatment with MeJA led to a further increase in total phenolic content. The highest values were recorded for 50 and 100 µM MeJA (11.33 and 11.38 mg/g DW, respectively), nearly two-fold higher than in the control (6.27 mg/g DW). At the same time, treatment with the highest elicitor concentration (200 µM MeJA) for 7 days resulted in a decrease in total phenolic content compared with lower concentrations, although the values remained higher than those in the control.

Treatment with MeJA led to an increased flavonoid synthesis (Figure 3). On day 3, flavonoid levels increased compared with the control, while no significant differences were observed among the different MeJA concentrations. As observed for phenolic compounds, prolonged treatment resulted in greater accumulation of these metabolites. The flavonoid content increased sharply after 7 days of treatment with 50 µM MeJA, but as the elicitor concentration increased, their levels decreased.

The water-soluble antioxidant content increased under the influence of MeJA (Figure 3). The highest value was recorded in plants treated with 100 µM MeJA for 3 days—10.72 µmol/g DW. However, on day 7, their content decreased compared with day 3, although it remained higher than in the control. The highest level of lipid-soluble antioxidant metabolites was also obtained with 100 µM MeJA treatment for 3 days—1.2 µmol/g DW (Figure 3). On day 7, the highest content of lipid-soluble metabolites was observed in plants grown on medium containing 50 µM MeJA—0.75 µmol/g DW. For the other two tested concentrations, 100 and 200 µM MeJA, a decrease in LS-AOM content was observed on the 7th day of treatment compared to the control.

### 3.4. Effect of MeJA on Total Antioxidant Activity

**Figure 4:**
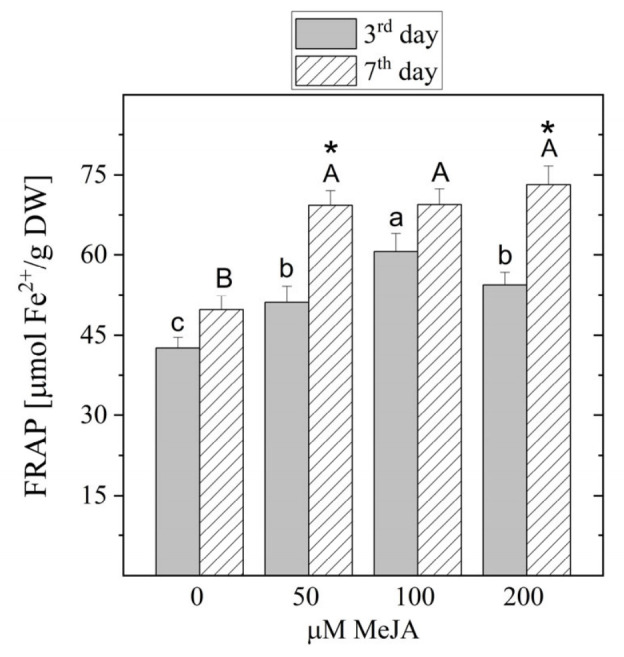
Antioxidant potential (FRAP) in *A. montana* plantlets elicited with MeJA applied at different concentrations (0, 50, 100, and 200 µM) on the 3rd and 7th day of the treatment. Values are means ± SE, *n* = 3; different letters indicate significant differences assessed by the Fisher LSD test (*p* ≤ 0.05) after performing ANOVA one-way analysis. We used the letter ‘a’ or “A” for the highest data value and descended to the next for lower data values. The statistical analysis of the 3rd day (lowercase) and 7th day (uppercase) was performed separately. The (*) indicates a significant difference between 3rd and 7th day in the same studied MeJA concentration.

In vitro plants cultivated for 3 days on nutrient media containing MeJA increased their antioxidant potential, as measured by the FRAP method. The best results were obtained in plants treated with 100 µM MeJA, with a 42% increase compared to the control. For 50 and 200 µM MeJA, the increase amounted to 20 and 29%, respectively. Ferric reducing antioxidant power values (FRAP) gradually increased (by 41%, 41%, and 49%, respectively) with increasing concentrations of MeJA in plants collected on the 7th day of treatment (Figure 4).

### 3.5. Caffeoylquinic Acids Content in A. montana After Elicitation with MeJA

**Figure 5:**
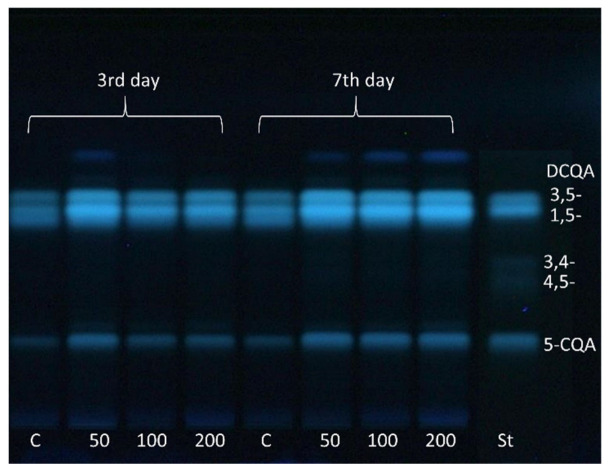
High-performance thin-layer chromatography (HPTLC) of methanolic extracts from control (C) plants, plants treated with various concentrations of MeJA (50, 100, and 200 μM) on the 3rd and 7th day of treatment, and a standard mixture (St).

**Figure 6:**
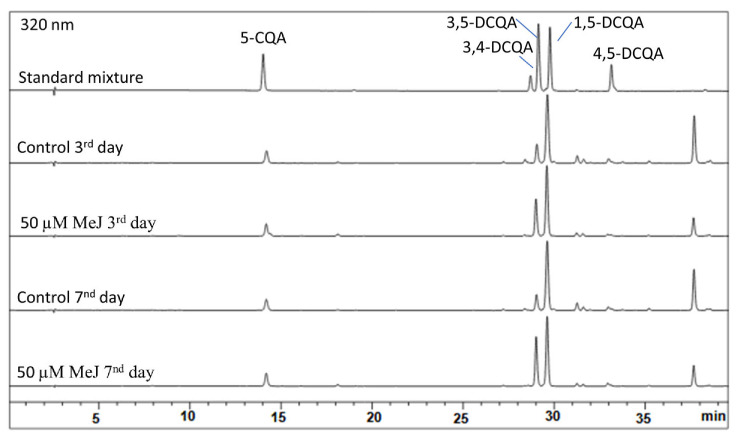
HPLC chromatogram of the standard mixture, control sample, and sample treated with 50 µM MeJA at 320 nm.

A preliminary analysis of the methanolic extracts from in vitro plants treated with MeJA, conducted using high-performance thin-layer chromatography (HPTLC) and authentic standards, showed the presence of chlorogenic acid (5-CQA), and 3,5- and 1,5-dicaffeoylquinic acids (DCQAs) and traces of 3,4- and 4,5-DCQA (Figure 5 and Figure 6). Further, these acids were used to assess the effect of MeJA on the metabolic profile of *A. montana*. For quantitative analysis, high-performance liquid chromatography with photodiode array detection (HPLC-PDA) was applied.

Quantitative analysis of caffeoylquinic acids showed that 1,5-dicaffeoylquinic acid was the predominant compound in the extracts (1.431–6.207 mg/g DW), followed by 3,5-dicaffeoylquinic acid (0.291–4.049 mg/g DW), chlorogenic acid (0.384–1.848 mg/g DW), 4,5-dicaffeoylquinic acid (0.073–0.267 mg/g DW), and 3,4-dicaffeoylquinic acid (0.044–0.085 mg/g DW) (Table 3, Figure 5). The content of all caffeoylquinic acids increased in response to MeJA treatment. On the third day of treatment with 50 µM MeJA, the total amount of caffeoylquinic acids increased more than four-fold (9.820 mg/g DW compared to 2.240 mg/g DW in the control). At 100 and 200 µM of MeJA, a decrease in caffeoylquinic acid content was observed, although the levels of these metabolites remained higher than those in control plants.

On the seventh day of MeJA treatment, higher contents of caffeoylquinic acids were recorded for all tested MeJA concentrations compared to the levels measured on the third day. The best results were observed at 100 µM MeJA, where the total content of caffeoylquinic acids reached 12.373 mg/g DW (3.8 times higher than the control).

## 4. Discussion

To assess the effect of MeJA on the in vitro growth of *A. montana*, fresh biomass, plant height, and the average number of shoots after cultivation were measured. The selected MeJA concentrations were based on previous reports showing efficient induction of phenolic biosynthesis in plant in vitro cultures within the 50–200 µM range, while minimizing severe inhibitory effects on biomass [45,46,47]. In addition, MeJA elicitation in plant in vitro cultures is commonly performed for periods ranging from 24 h to several days, depending on the culture system and target metabolites. Our preliminary investigation indicated that prolonged exposure to MeJA results in the suppression of plant development and necrosis. Therefore, this growth regulator was applied for 3 and 7 days to evaluate its effect on growth, antioxidant activity, and phenolic accumulation. According to the obtained results, the concentration and duration of treatment are essential for the effect of the applied elicitor on arnica growth. Seven-day treatment negatively affected the investigated morphometric parameters, with the lowest values recorded at 200 µM MeJA. In the scientific literature, both positive and negative effects of MeJA application on in vitro plant growth have been reported [48]. Generally, the effect of MeJA depends on the plant species, the concentration used, and treatment duration [49]. The optimal concentration of MeJA varies widely among different plant species. For example, treatment with 1 μM MeJA led to a 10.89% increase in the fresh weight of *Brassica oleracea* [50]; 2 and 5 μM MeJA increased plant height in *Artemisia annua* [51]; 25–75 μM MeJA induced direct organogenesis and stimulated growth of *Hemidesmus indicus* [52]; 50 and 200 μM MeJA increased biomass of callus cultures of *Talinum paniculatum* [53]. The best concentration of MeJA for plant height, fresh weight, leaf and shoot numbers, and callogenesis in in vitro cultures of *Gynura pseudochina* was determined to be 150 µM, whereas 75 µM of MeJA was identified as optimal for root growth [54]. However, in some plant species, at unfavorable concentrations, MeJA inhibits plant growth. Treatment with 100 µM MeJA was reported to reduce the biomass of root cultures of *Polygonum multiflorum* and *Echinacea purpurea* by 5.8% and 22.97%, respectively [55]. Biomass and growth of callus, shoot, and root cultures of *Echinacea purpurea* were reduced after 50, 100, 150, and 200 μM MeJA treatment [56]. MeJA reduces biomass accumulation in various organ cultures grown under in vitro conditions, such as root cultures of *Eleutherococcus koreanum* [57], *Echinacea pallida* and *Echinacea purpurea* [58], and embryos of *Eleutherococcus sessiliflorus* [59]. According to Noir et al. [60], MeJA contributes to driving two possibly independent strategies to reschedule the energy between growth and stress responses. In *Arabidopsis thaliana*, the inhibited leaf growth resulting from 50 μM MeJA treatment was manifested by a reduction in cell number and cell size [60]. Analysis of gene expression of the *Arabidopsis* genome showed that MeJA inhibited the activation of the M phase genes, thus cells were arrested in the G2 phase of the cell cycle [61]. It has been established that in the plant cell, MeJA is demethylated to jasmonate (JA) and exerts its effect through binding to the JA receptor COI1 [60]. The inhibition of cell division (G2/M transition) by MeJA may represent an adaptation to redirect cellular resources towards rapid restoration of homeostasis after the release of the stress signal. Alternatively, blocking the cell cycle before completion of DNA replication (G1/S) may be a mechanism for additional resource conservation. MeJA may contribute to the arrest of DNA replication by disrupting the pre-replicative complex assembly [60]. It has been reported that MeJA primarily induces the expression of genes involved in jasmonate biosynthesis, and subsequently suppresses cell cycle-related genes while activating genes of the phenylpropanoid pathway [62,63]. These findings show the presence of a MeJA-regulated COI1-dependent growth control mechanism, allowing the redirection of metabolism towards increased production of secondary metabolites related to defense [63]. Krishnan et al. [64] reported that MeJA inhibited the growth of shoot, callus, and cell suspension cultures of *Centella asiatica*, while promoting the biosynthesis of asiaticoside (in shoot and callus cultures) and asiatic acid (in callus cultures).

Antioxidant enzyme analysis in *A. montana* plantlets revealed a complex, dose-dependent response to MeJA. Specifically, MeJA exerted a stimulatory effect on SOD and partially on APX, while inhibiting CAT and GPX activities. Regarding H_2_O_2_ scavenging, APX was the only enzyme that showed increased activity at 50 and 100 µM MeJA compared with the control. However, its activity dropped sharply at the highest concentration. The elevated activity of SOD (which converts O_2_^•−^ into H_2_O_2_), combined with the suppressed activities of H_2_O_2_-scavenging enzymes (CAT, APX, and GPX) at 200 µM MeJA, likely led to excessive H_2_O_2_ accumulation and oxidative burden. This onset of severe oxidative stress is further supported by the visible necrosis observed at 200 µM MeJA.

Numerous studies have shown that MeJA enhances the activity of antioxidant enzymes such as APX, GPX, CAT, and SOD [65]. However, the literature also contains data of an inhibitory effect on some of these enzymes. Some authors have observed a decrease in catalase activity following MeJA treatment, as reported for in vitro cultures of *Hypericum perforatum* [66] and *Stevia rebaudiana* Bertoni [18]. *Polyscias filicifolia* (*Araliaceae* family) shoot cultures elicited with MeJA showed a decrease in CAT activity and an increase in GPX activity [67]. Increased activities of SOD and GPX have been reported after MeJA treatment of root cultures of *Panax ginseng* and *Panax quinquefolium* [68]. SOD and CAT activities were significantly reduced in roots of *Cnidium officinale* treated with MeJA, whereas GPX and APX activities were approximately 1.33 and 1.48 times higher than the control, respectively [55]. MeJA at a concentration of 10 μM effectively alleviated the adverse effects of low-temperature stress on the growth of *Solanum lycopersicum.* This protective effect may be attributed to reduced H_2_O_2_ accumulation, enhanced activities of antioxidant enzymes (SOD, CAT, and APX), and improved photosynthetic performance [65]. These data show that the effect of MeJA on enzymatic antioxidant activity largely depends on plant species, plant organs, and specific growing conditions.

SOD is the first line of antioxidant enzymes in plants. It catalyzes the dismutation of superoxide radicals (O_2_^•−^) to H_2_O_2_ [69]; APX is involved in H_2_O_2_ scavenging in chloroplasts through the water-water cycle and the ascorbate–glutathione (ASH-GSH) cycle and shows a higher affinity to hydrogen peroxide than catalase [70]; GPX is known to play a role in the biosynthesis of lignin and defense against biotic stresses by consuming H_2_O_2_. The polymerization of monolignols into lignin involves an oxidative mechanism mediated by oxidative enzymes, such as peroxidases, in the presence of hydrogen peroxide. GPX (class III peroxidases) uses hydrogen peroxide (H_2_O_2_) to oxidize monolignols into phenoxy radicals, which then polymerize to form lignin in the cell wall. This process strengthens the cell wall, improves tissue mechanical stability, and enhances protection against pathogens [71]. In our study, the activity of class III peroxidases, which are typically induced under biotic stress conditions, decreased following MeJA treatment. This suggests that these enzymes may not play a central role in ROS detoxification in response to MeJA, an abiotic elicitor.

Despite the reduced activity of H_2_O_2_-detoxifying enzymes, moderate ROS accumulation, particularly of H_2_O_2_, may serve a signaling role by upregulating the biosynthesis of stress-induced secondary metabolites (e.g., phenolics, flavonoids, and ascorbic acid), thereby contributing to long-term defense responses [55,72,73].

MeJA supplementation of the Murashige and Skoog nutrient medium at all tested concentrations led to increased total phenolic and flavonoid contents in *A. montana* plantlets as early as the third day of treatment, with levels continuing to increase until the seventh day. This suggests that MeJA stimulates the production of these antioxidant compounds over time. Our findings are consistent with the results reported for other plant species. It has been found that MeJA applied at concentrations of 100 and 150 μM increases the accumulation of total phenolics and caffeic acid derivatives in in vitro cultures of callus, aerial parts, and roots of *Echinacea purpurea* [56]. Enhanced total phenolic content was observed in micropropagated *Stevia rebaudiana* plants treated with 100 μM MeJA [18]. Cultivation of *Eryngium planum* L. shoot cultures in liquid MS medium containing 1 mg/L BAP and 0.1 mg/L IAA in the presence of 100 μM MeJA for 48 h led to approximately a 4.5-fold increase in rosmarinic, chlorogenic, and caffeic acids compared to the control [74]. An increased production of phenolic acids in shoot cultures of *Salvia virgata* Jacq. and *Exacum affine* Balf. has also been observed following MeJA application [75,76]. Wang et al. [66] reported that MeJA affects cell growth and flavonoid production in cell suspensions of *Hypericum perforatum*, with the highest flavonoid production (280 mg/L) achieved following treatment with 100 μM MeJA for 15 days. The authors found that MeJA treatment enhances phenylalanine ammonia-lyase (PAL) activity, thereby stimulating flavonoid biosynthesis. Increased total phenolic and flavonoid contents following MeJA application have also been reported in proliferated plants of the endemic species *Haplophyllum virgatum* var. *virgatum* [77]. Short-term MeJA treatments were shown to rapidly increase pyrethrin accumulation in *Tanacetum cinerariifolium,* although these effects were transient. In contrast, long-term treatment with low MeJA concentrations promoted glandular trichome density, resulting in more stable levels of secondary metabolites [78,79]. The authors suggested that with long-term exposure to MeJA, seedlings gradually adapt to exogenous hormonal signals, leading to morphological changes that allow for increased pyrethrin production.

Assessing antioxidant compounds in plants is essential for understanding their contribution to plant development and their role in total antioxidant defense against oxidative stress. Quantitative assessment of water-soluble and lipid-soluble antioxidants (including polyphenols, flavonoids, vitamins, and beta-carotene) is important for evaluating their contribution to the overall antioxidant capacity involved in free radical scavenging. MeJA has been shown to stimulate the production of water-soluble antioxidants (total phenolics, flavonoids, ascorbate) in in vitro cultures through activation of the phenylpropanoid pathway across different plant systems [80]. In parallel, increased content of lipid-soluble antioxidants (e.g., carotenoids and tocopherols), associated with MeJA-induced oxidative stress and defense responses, has been reported in organ and shoot cultures, although the effect varied according to species and culture conditions [81,82]. Despite the higher phenolic levels in *A. montana* plantlets with the increasing MeJA treatment time (7th day), the content of water-soluble antioxidants was lower than that of the short-term treatment (3rd day). This discrepancy could be due to the inclusion of other hydrophilic secondary metabolites with antioxidant potential, such as vitamin C, certain amino acids, and reducing sugars, in addition to phenol-type molecules when evaluating water-soluble antioxidants. These compounds may be negatively affected by prolonged MeJA treatment, resulting in a significant decrease in their content over time. In our previous study, the content of water-soluble antioxidant metabolites increased after yeast extract treatment of in vitro shoots, while lipid-soluble antioxidants were enhanced in response to salicylic acid treatment [33].

The total antioxidant activity of arnica extracts, as determined by the FRAP assay, increased after three days of treatment with MeJA at all tested concentrations, with a further increase observed after prolonged treatment (7 days). Our results are in agreement with previously published data demonstrating the role of jasmonates in increasing the antioxidant capacity of a wide range of plant species, including *Salvia tebesana* Bunge [46], *Ruta graveolens* L. [83], *Portulaca oleracea* L. [73], etc.

Elicitation with MeJA is a promising strategy for increasing phenolic compound production. MeJA applied at a concentration of 200 µM effectively stimulated the synthesis of caffeoylquinic acids in cell suspension cultures of *Gardenia jasminoides*, increasing their content by 21.7-fold compared to the control [84]. The amount of phenolic acids, including chlorogenic acid, increased after treatment of *Aster scaber* hairy roots with 100 μM MeJA [85]. Caffeoylquinic acid production increased approximately threefold in hairy root cultures of *Cichorium intybus* [22] and by 4.4-fold in hairy roots of *Ficus carica* L. following MeJA application [86].

Quantitative analysis of individual caffeoylquinic acids revealed that 1,5-DCQA was the dominant compound in the analyzed *A. montana* plantlet samples. Similar results have been reported by other authors, who found that 1,5-dicaffeoylquinic acids are the main components in the flowers and aerial parts of *A. montana* [25,87]. Other studies have shown that 3,5-DCQA is the primary phenolic acid in samples from both cultivated and wild-grown plants [88,89], followed by 5-CQA and 1,5-DCQA, with 4,5-DCQA detected in the lowest amounts.

MeJA treatment increased total phenols, flavonoids, and caffeoylquinic acids, with maximal accumulation observed after 7 days. In contrast, our previous analysis of the same plants revealed only a slight transient increase in sesquiterpene lactone content at 50 and 100 µM MeJA after 3 days, whereas 200 µM reduced sesquiterpene lactone accumulation [30]. After 7 days, lactone levels remained unchanged at 50 and 100 µM, and decreased at 200 µM compared to the control. These findings suggest that prolonged MeJA exposure may redirect signaling and metabolic flux toward the phenylpropanoid pathway, although molecular validation is still required.

## 5. Conclusions

Stem explants from in vitro germinated plants were successfully used to induce direct organogenesis and micropropagation in *A. montana* L. Numerous clonal plants were obtained during cultivation on MS agar-based nutrient medium supplemented with 0.5 mg/L BAP. MeJA addition to the culture medium inhibited plant growth and development when applied for seven days. However, it stimulated the synthesis of caffeoylquinic acids, total phenolics, and flavonoids, as well as water- and lipid-soluble antioxidants. The antioxidant enzymes superoxide dismutase and ascorbate peroxidase showed increased activity, while catalase and guaiacol peroxidase activity decreased in in vitro plants treated with MeJA.

Our results suggest that MeJA treatment redirects *A. montana* resources from growth toward defense responses, as reflected by reduced growth, increased accumulation of antioxidant metabolites, and higher activity of key antioxidant enzymes, including SOD and APX.

This study provides a foundation for future, more detailed studies on the mechanisms regulating the biosynthesis of biologically active substances under the influence of MeJA and the associated changes in gene expression induced by this elicitor.

## Figures and Tables

**Table 1:** Concentration range, regression equation, correlation coefficient (R^2^), limit of detection (LOD) and limit of quantification (LOQ) of caffeoylquinic acids.

Compound	Concentration Range (mg/mL)	Regression Equation	R^2^	LOD/LOQ (mg/mL)
5-CQA	0.019–0.305	y = 4794840x − 650.239	0.9999	0.003/0.010
3,4-DCQA	0.005–0.083	y = 6844290x − 2226.57	0.9999	0.002/0.005
3,5-DCQA	0.019–0.308	y = 8033360x + 4885.41	0.9999	0.002/0.007
1,5-DCQA	0.022–0.355	y = 6856150x − 15457.9	0.9998	0.011/0.033
4,5-DCQA	0.008–0.135	y = 7409690x + 6581.95	0.9999	0.002/0.007

**Table 2:** Morphometric parameters of in vitro propagated *A. montana* after elicitation with MeJA.

Treatment	Number of Shoots	Shoot Height	FW
	per Explant	cm	g
3rd day			
0 µM MeJA	4.4 ± 0.30 ^a^	1.61 ± 0.09 ^a^	0.56 ± 0.02 ^a^
50 µM MeJA	4.1 ± 0.31 ^a^	1.40 ± 0.07 ^ab^	0.48 ± 0.04 ^ab^
100 µM MeJA	4.2 ± 0.29 ^a^	1.53 ± 0.14 ^ab^	0.51 ± 0.03 ^ab^
200 µM MeJA	3.9 ± 0.26 ^a^	1.31 ± 0.09 ^b^	0.44 ± 0.03 ^b^
LSD	0.82	0.24	0.10
7th day			
0 µM MeJA	4.65 ± 0.30 ^a^	1.91 ± 0.06 ^a^	0.62 ± 0.03 ^a^
50 µM MeJA	4.0 ± 0.20 ^ab^	1.58 ± 0.04 ^b^	0.54 ± 0.04 ^a^
100 µM MeJA	3.8 ± 0.27 ^b^	1.50 ± 0.07 ^b^	0.43 ± 0.04 ^b^
200 µM MeJA	3.5 ± 0.25 ^b^	1.30 ± 0.05 ^c^	0.37 ± 0.03 ^b^
LSD	0.74	0.17	0.10

*Data are presented as mean values ± standard error (SE). Different letters indicate significant differences determined by Fisher’s LSD test (p ≤ 0.05) following one-way ANOVA. The letter ‘a’ denotes the highest value, with subsequent letters assigned to progressively lower values.*

**Table 3:** Caffeoylquinic acid content [mg/g DW] in *A. montana* treated with various MeJA concentrations in vitro.

Treatment	5-CQA	3,4-DCQA	3,5-DCQA	1,5-DCQA	4,5-DCQA	Total
3rd day						
control	0.384 ± 0.006 ^d^	0.061 ± 0.006 ^b^	0.291 ± 0.012 ^d^	1.431 ± 0.008 ^d^	0.073 ± 0.003 ^c^	2.240 ± 0.036 ^d^
50 MeJA	1.480 ± 0.006 ^a^	0.085 ± 0.001 ^a^	2.731 ± 0.011 ^a^	5.39 ± 0.017 ^a^	0.135 ± 0.003 ^a^	9.820 ± 0.032 ^a^
100 MeJA	0.799 ± 0.023 ^b^	0.066 ± 0.001 ^b^	1.434 ± 0.008 ^b^	3.066 ± 0.009 ^b^	0.084 ± 0.002 ^b^	5.450 ± 0.025 ^b^
200 MeJA	0.751 ± 0.006 ^c^	0.046 ± 0.002 ^c^	1.050 ± 0.035 ^c^	2.607 ± 0.019 ^c^	0.070 ± 0.002 ^c^	4.523 ± 0.008 ^c^
LSD	0.041	0.010	0.067	0.047	0.009	0.089
7th day						
control	0.531 ± 0.006 ^c^	0.051 ± 0.006 ^a,b^	0.383 ± 0.008 ^d^	2.192 ± 0.017 ^c^	0.087 ± 0.006 ^c^	3.243 ± 0.044 ^d^
50 MeJA	1.848 ± 0.018 ^a^	0.057 ± 0.000 ^a^	3.563 ± 0.038 ^b^	6.162 ± 0.065 ^a^	0.267 ± 0.009 ^a^	11.898 ± 0.127 ^b^
100 MeJA	1.833 ± 0.022 ^a^	0.051 ± 0.001 ^a,b^	4.049 ± 0.058 ^a^	6.207 ± 0.075 ^a^	0.232 ± 0.017 ^a,b^	12.373 ± 0.065 ^a^
200 MeJA	1.753 ± 0.017 ^b^	0.044 ± 0.002 ^b^	2.932 ± 0.049 ^c^	5.702 ± 0.048 ^b^	0.196 ± 0.011 ^b^	10.626 ± 0.059 ^c^
LSD	0.051	0.011	0.138	0.182	0.038	0.269

*Data are presented as mean values ± standard error (SE). Different letters indicate significant differences determined by Fisher’s LSD test (p ≤ 0.05) following one-way ANOVA. The letter ‘a’ denotes the highest value, with subsequent letters assigned to progressively lower values.*

## Data Availability

All data are comprised in the manuscript.

## Abbreviations

The following abbreviations are used in this manuscript:

MeJA	Methyl jasmonate
MS	Murashige and Skoog nutrient medium
BAP	6-Benzylaminopurine
IAA	Indole-3-acetic acid
SOD	Superoxide dismutase
APX	Ascorbate peroxidase
CAT	Catalase
GPX	Guaiacol peroxidase
WS-AOM	Water-Soluble Antioxidants
LS-AOM	Lipid-Soluble Antioxidants
CQAs	Caffeoylquinic acids
TPC	Total phenolic content
TFC	Total flavonoid content
FRAP	Ferric-reducing antioxidant power

## References

[1] Kriplani P., Guarve K., Baghael U.S. (2017). *Arnica montana* L.—A plant of healing: Review. J. Pharm. Pharmacol..

[2] Gyawali N., Rayamajhi A., Karki D., Pokhrel T., Adhikari A., Devkota H.P., Aftab T. (2022). *Arnica montana* L.: Traditional Uses, Bioactive Chemical Constituents, and Pharmacological Activities. Medicinal Plants of the Asteraceae Family.

[3] Vera M., Mora G., Rodríguez-Guitián M.A., Blanco A., Casanova A., Real C., Romero R., Bouza C. (2020). Living at the edge: Population differentiation in endangered *Arnica montana* from NW Iberian Peninsula. Plant Syst. Evol..

[4] Atanasov A.G., Waltenberger B., Pferschy-Wenzig E.-M., Linder T., Wawrosch C., Uhrin P., Temml V., Wang L., Schwaiger S., Heiss E.H. (2015). Discovery and resupply of pharmacologically active plant-derived natural products: A review. Biotechnol. Adv..

[5] Isah T., Umar S., Mujib A., Sharma M.P., Rajasekharan P.E., Zafar N., Frukh A. (2018). Secondary metabolism of pharmaceuticals in the plant in vitro cultures: Strategies, approaches, and limitations to achieving higher yield. Plant Cell Tissue Organ Cult..

[6] Wawrosch C., Zotchev S.B. (2021). Production of bioactive plant secondary metabolites through in vitro technologies—Status and outlook. Appl. Microbiol. Biotechnol..

[7] Conchou O., Nichterlein K., Vömel A. (1992). Shoot tip culture of *Arnica montana* for micropropagation. Planta Medica.

[8] Surmackz-Magdziak A., Sugier D. (2012). In vitro propagation of *Arnica montana* L.: An endangered herbal species of great importance to medicine. Acta Sci. Pol. Hortorum Cultus.

[9] Petrova M., Zayova E., Geneva M., Dimitrova L., Vitkova A., Stanilova M. (2021). Multiplication and conservation of threatened medicinal plant *Arnica montana* L. by in vitro techniques. Agric. Consp. Sci..

[10] Zhao J., Davis L.C., Verpoorte R. (2005). Elicitor signal transduction leading to production of plant secondary metabolites. Biotechnol. Adv..

[11] Kohli S.K., Handa N., Sharma A., Gautam V., Arora S., Bhardwaj R., Wijaya L., Alyemeni M.N., Ahmad P. (2018). Interaction of 24-Epibrassinolide and Salicylic Acid Regulates Pigment Contents, Antioxidative Defense Responses, and Gene Expression in *Brassica juncea* L. Seedlings under Pb Stress. Environ. Sci. Pollut. Res..

[12] Thakur M., Bhattacharya S., Khosla P.K., Puri S. (2019). Improving Production of Plant Secondary Metabolites through Biotic and Abiotic Elicitation. J. Appl. Res. Med. Aromat. Plants.

[13] Guru A., Dwivedi P., Kaur P., Pandey D.K. (2022). Exploring the role of elicitors in enhancing medicinal values of plants under in vitro condition. S. Afr. J. Bot..

[14] Ahmed S.A., Baig M.M.V. (2014). Biotic elicitor enhanced production of psoralen in suspension cultures of *Psoralea corylifolia* L.. Saudi J. Biol. Sci..

[15] Giri C.C., Zaheer M. (2016). Chemical elicitors versus secondary metabolite production in vitro using plant cell, tissue and organ cultures: Recent trends and a sky eye view appraisal. Plant Cell Tissue Organ Cult..

[16] Portu J., López R., Santamaría P., Garde-Cerdán T. (2017). Elicitation with methyl jasmonate supported by precursor feeding with phenylalanine: Effect on Garnacha grape phenolic content. Food Chem..

[17] Gabr A.M.M., Ghareeb H., El Shabrawi H.M., Smetanska I., Bekheet S.A. (2016). Enhancement of silymarin and phenolic compound accumulation in tissue culture of Milk thistle using elicitor feeding and hairy root cultures. J. Genet. Eng. Biotechnol..

[18] Moharramnejad S., Azam A.T., Panahandeh J., Dehghanian Z., Ashraf M. (2019). Effect of methyl jasmonate and salicylic acid on in vitro growth, stevioside production, and oxidative defense system in *Stevia rebaudiana*. Sugar Tech.

[19] Ravazzolo L., Ruperti B., Frigo M., Bertaiola O., Pressi G., Malagoli M., Quaggiotti S. (2022). *C3H* Expression Is Crucial for Methyl Jasmonate Induction of Chicoric Acid Production by *Echinacea purpurea* (L.) Moench Cell Suspension Cultures. Int. J. Mol. Sci..

[20] Ahlawat S., Saxena P., Alam P., Wajid S., Abdin M.Z. (2014). Modulation of artemisinin biosynthesis by elicitors, inhibitor, and precursor in hairy root cultures of *Artemisia annua* L.. J. Plant Interact..

[21] Shabani L., Ehsanpour A., Asghari G., Emami J. (2009). Glycyrrhizin production by in vitro cultured *Glycyrrhiza glabra* elicited by methyl jasmonate and salicylic acid. Russ. J. Plant Physiol..

[22] Bernard G., Dos Santos H.A., Etienne A., Samaillie J., Neut C., Sahpaz S., Hilbert J.-L., Gagneul D., Jullian N., Tahrioui A. (2020). MeJA elicitation of chicory hairy roots promotes efficient increase of 3,5-diCQA accumulation, a potent antioxidant and antibacterial molecule. Antibiotics.

[23] Sun L., Di D.W., Li G., Li Y., Kronzucker H.J., Shi W. (2020). Transcriptome analysis of rice (*Oryza sativa* L.) in response to ammonium resupply reveals the involvement of phytohormone signaling and the transcription factor OsJAZ9 in reprogramming of nitrogen uptake and metabolism. J. Plant Physiol..

[24] Nichterlein K., Bajaj Y.P.S. (1995). Arnica Montana (Mountain Arnica): In Vitro Culture and the Production of Sesquiterpene Lactones and Other Secondary Metabolites. Medicinal and Aromatic Plants VIII.

[25] Jaiswal R., Kuhnert N. (2011). Identification and characterization of two new derivatives of chlorogenic acids in Arnica (*Arnica montana* L.) flowers by high-performance liquid chromatography/tandem mass spectrometry. J. Agric. Food Chem..

[26] Kimel K., Krauze-Baranowska M., Godlewska S., Pobłocka-Olech L. (2020). HPLC-DAD-ESI/MS comparison of the chemical composition of flowers from two *Arnica* species grown in Poland. Herba Pol..

[27] Jaiswal R., Kiprotich J., Kuhnert N. (2011). Determination of the hydroxycinnamate profile of 12 members of the Asteraceae family. Phytochemistry.

[28] Alcázar Magaña A., Kamimura N., Soumyanath A., Stevens J.F., Maier C.S. (2021). Caffeoylquinic acids: Chemistry, biosynthesis, occurrence, analytical challenges, and bioactivity. Plant J..

[29] Parafiniuk A., Kromer K., Fleszar M.G., Wróblewska K., Wiśniewski J.Ł., Gamian A. (2025). Impact of elicitors and light on biosynthesis of sesquiterpene lactones in tissue culture of *Arnica montana* and its variety Arbo. Front. Plant Sci..

[30] Sozoniuk M., Trendafilova A., Mishev K., Geneva M., Miladinova-Georgieva K., Ivanova V., Dimitrova L., Dimitrova M., Petrova M. (2026). Sesquiterpene lactones in micropropagated *Arnica montana* shoots after elicitation—Insights into metabolite accumulation and transcriptional regulation. Sci. Rep..

[31] Rasouli H., Farzaei M.H., Khodarahmi R. (2017). Polyphenols and their benefits: A review. Int. J. Food Prop..

[32] Kanthaliya B., Joshi A., Arora J., Alqahtani M.D., Abd_Allah E.F. (2023). Effect of Biotic Elicitors on the Growth, Antioxidant Activity and Metabolites Accumulation in In Vitro Propagated Shoots of *Pueraria tuberosa*. Plants.

[33] Petrova M., Geneva M., Trendafilova A., Miladinova-Georgieva K., Dimitrova L., Sichanova M., Nikolova M., Ivanova V., Dimitrova M., Sozoniuk M. (2025). Antioxidant Capacity and Accumulation of Caffeoylquinic Acids in *Arnica montana* L. In Vitro Shoots After Elicitation with Yeast Extract or Salicylic Acid. Plants.

[34] Murashige T., Skoog F. (1962). A revised medium for rapid growth and bio assays with tobacco tissue cultures. Physiol. Plant..

[35] Bradford M.M. (1976). A rapid and sensitive method for the quantitation of microgram quantities of protein utilizing the principle of protein-dye binding. Anal. Biochem..

[36] Giannopolitis C.N., Ries S.K. (1977). Superoxide dismutases I. Occurrence in higher plants. Plant Physiol..

[37] Beers R.F., Sizer I.W. (1952). A spectrophotometric method for measuring the breakdown of hydrogen peroxide by catalase. J. Biol. Chem..

[38] Nakano Y., Asada K. (1987). Purification of ascorbate peroxidase in spinach chloroplasts: Its inactivation in ascorbate-depleted medium and reactivation by monodehydroascorbate radical. Plant Cell Physiol..

[39] Urbanek H., Kuźniak-Gębarowska E., Herka K. (1991). Elicitation of defence responses in bean leaves by *Botrytis cinerea* polygalacturonase. Acta Physiol. Plant..

[40] Pfeffer H., Dannel F., Römheld V. (1998). Are there connection between phenol metabolism, ascorbate metabolism and membrane integrity in leaves of boron-deficient sunflower plants?. Physiol. Plant..

[41] Jia Z., Tang M., Wu J. (1999). The determination of flavonoid contents in mulberry and their scavenging effects on superoxide radical. Food Chem. J..

[42] Prieto P., Pineda M., Aguilar M.A. (1999). Spectrophotometric quantitation of antioxidant capacity through the formation of a phosphomolybdenum complex: Specific application to the determination of vitamin E. Anal. Bioch..

[43] Benzie I., Strain J. (1996). The ferric reducing ability of plasma (FRAP) as a measure of “antioxidant power”: The FRAP assay. Anal. Bioch..

[44] Ivanova V., Nedialkov P., Dimitrova P., Paunova-Krasteva T., Trendafilova A. (2024). *Inula salicina* L.: Insights into Its Polyphenolic Constituents and Biological Activity. Pharmaceuticals.

[45] Ben Romdhane A., Chtourou Y., Sebii H., Baklouti E., Nasri A., Drira R., Maalej M., Drira N., Rival A., Fki L. (2022). Methyl jasmonate induces oxidative/nitrosative stress and the accumulation of antioxidant metabolites in *Phoenix dactylifera* L.. Biotechnol. Lett..

[46] Shoja A.A., Çirak C., Ganjeali A., Cheniany M. (2022). Stimulation of Phenolic Compounds Accumulation and Antioxidant Activity in in Vitro Culture of *Salvia tebesana* Bunge in Response to Nano-TiO_2_ and Methyl Jasmonate Elicitors. Plant Cell Tissue Organ Cult. PCTOC.

[47] Amani S., Mohebodini M., Khademvatan S., Jafari M., Kumar V. (2024). Modifications in Gene Expression and Phenolic Compounds Content by Methyl Jasmonate and Fungal Elicitors in *Ficus carica*. Cv. Siah Hairy Root Cultures. BMC Plant Biol..

[48] Singh B. (2024). A review on the effects of jasmonates on plants grown under in vitro conditions. Afr. J. Biomed. Res..

[49] Đurić M., Subotić A., Prokić L., Trifunović-Momčilov M., Milošević S. (2023). Alterations in Physiological, Biochemical, and Molecular Responses of *Impatiens walleriana* to Drought by Methyl Jasmonate Foliar Application. Genes.

[50] Sirhindi G., Mushtaq R., Gill S.S., Sharma P., Abd_Allah E.F., Ahmad P. (2020). Jasmonic acid and methyl jasmonate modulate growth, photosynthetic activity and expression of photosystem II subunit genes in *Brassica oleracea* L.. Sci. Rep..

[51] Alam P., Albalawi T.H. (2020). In vitro alteration of artemisinin biosynthesis in *Artemisia annua* L during treatment with methyl jasmonate. Trop. J. Pharm. Res..

[52] Nandy S., Hazra A.K., Pandey D.K., Ray P., Dey A. (2021). Elicitation of Industrially Promising Vanillin Type Aromatic Compound 2-Hydroxy 4-Methoxy Benzaldehyde (MBAlD) Yield in the in-Vitro Raised Medicinal Crop *Hemidesmus indicus* (L) R. Br. by Methyl Jasmonate and Salicylic Acid. Ind. Crops Prod..

[53] Restiani R., Aditiyarini D., Barlin N. (2022). Effect of methyl Jasmonate on biomass and Saponin content in Javanese ginseng (*Talinum paniculatum* (Jacq.) Gaertn.) callus culture. Sch. Acad. J. Biosci..

[54] Anjalani T.R., Rasmi S.A., Rahayu A.E., Ramadhani M.R.N., Sholihah M.F., Puspaningtyas I., Datus Soleha I., Sari S., Rahmawati M., Nasori N. (2024). Methyl jasmonate stimulates growth and upregulates the expression of Phenylalanine Ammonia-Lyase (PAL) gene in *Gynura pseudochina* in vitro micropropagation. Biodiversitas J. Biol. Divers..

[55] Ho T.-T., Murthy H.N., Park S.-Y. (2020). Methyl Jasmonate Induced Oxidative Stress and Accumulation of Secondary Metabolites in Plant Cell and Organ Cultures. Int. J. Mol. Sci..

[56] Demirci T. (2022). Determination of secondary metabolite production efficiency in *Echinacea purpurea* callus, shoot, and root in vitro cultures with methyl jasmonate applications. Acta Physiol. Plant..

[57] Lee E.J., Park S.Y., Paek K.Y. (2015). Enhancement strategies of bioactive compound production in adventitious root cultures of *Eleutherococcus koreanum* Nakai subjected to methyl jasmonate and salicylic acid elicitation through airlift bioreactors. Plant Cell Tissue Organ Cult..

[58] An D., Wu C.H., Wang M., Wang M., Chang G.N., Chang X.J., Lian M.L. (2022). Methyl jasmonate elicits enhancement of bioactive compound synthesis in adventitious root co-culture of *Echinacea purpurea* and *Echinacea pallida*. In Vitro Cell. Dev. Biol. Plant.

[59] Shohael A.M., Murthy H.N., Lee H.L., Hahn E.J., Paek K.Y. (2008). Increased eleutheroside production in *Eleutherococcus sessiliflorus* embryogenic suspension cultures with methyl jasmonate treatment. Biochem. Eng. J..

[60] Noir S., Bömer M., Takahashi N., Ishida T., Tsui T.-L., Balbi V., Shanahan H., Sugimoto K., Devoto A. (2013). Jasmonate Controls Leaf Growth by Repressing Cell Proliferation and the Onset of Endoreduplication While Maintaining a Potential Stand-By Mode. Plant Physiol..

[61] Pauwels L., Morreel K., De Witte E., Lammertyn F., Van Montagu M., Boerjan W., Inze D., Goossens A. (2008). Mapping methyl jasmonate-mediated transcriptional reprogramming of metabolism and cell cycle progression in cultured *Arabidopsis* cells. Proc. Natl. Acad. Sci. USA.

[62] Gumerova E.A., Akulov A.N., Rumyantseva N.I. (2015). Effect of methyl jasmonate on growth characteristics and accumulation of phenolic compounds in suspension culture of tartary buckwheat. Russ. J. Plant Physiol..

[63] Bömer M., O’Brien J.A., Pérez-Salamó I., Krasauskas J., Finch P., Briones A., Daudi A., Souda P., Tsui T.-L., Whitelegge J.P. (2018). COI1-dependent jasmonate signalling affects growth, metabolite production and cell wall protein composition in *Arabidopsis*. Ann. Bot..

[64] Krishnan M.L., Roy A., Bharadvaja N. (2019). Elicitation effect on the production of asiaticoside and asiatic acid in shoot, callus, and cell suspension culture of *Centella asiatica*. J. Appl. Pharm. Sci..

[65] Gul N., Masoodi K.Z., Ramazan S., Mir J.I., Aslam S. (2023). Study on the impact of exogenously applied methyl jasmonate concentrations on *Solanum lycopersicum* under low temperature stress. BMC Plant Biol..

[66] Wang J., Qian J., Yao L., Lu Y. (2015). Enhanced production of flavonoids by methyl jasmonate elicitation in cell suspension culture of Hypericum perforatum. Bioresour. Bioprocess..

[67] Śliwińska A., Naliwajski M.R., Pietrosiuk A., Sykłowska-Baranek K. (2021). In Vitro Response of *Polyscias filicifolia* (Araliaceae) Shoots to Elicitation with Alarmone–Diadenosine Triphosphate, Methyl Jasmonate, and Salicylic Acid. Cells.

[68] Ali M.B., Yu K.W., Hahn E.J., Paek K.Y. (2005). Differential responses of anti-oxidants enzymes, lipoxygenase activity, ascorbate content and the production of saponins in tissue cultured root of mountain *Panax ginseng* C.A. Mayer and *Panax quinquefolium* L. in bioreactor subjected to methyl jasmonate stress. Plant Sci..

[69] Szőllősi R., Ahmad A. (2014). Superoxide dismutase (SOD) and abiotic stress tolerance in plants: An overview. Oxidative Damage to Plants.

[70] Gill S.S., Tuteja N. (2010). Reactive Oxygen Species and Antioxidant Machinery in Abiotic Stress Tolerance in Crop Plants. Plant Physiol. Biochem..

[71] Pandey V.P., Awasthi M., Singh S., Tiwari S., Dwivedi U.N. (2017). A Comprehensive Review on Function and Application of Plant Peroxidases. Biochem. Anal. Biochem..

[72] Desikan R., Mackerness S.A.H., Hancock S.J.T., Neill S.J. (2001). Regulation of the Arabidopsis transcriptome by oxidative stress. Plant Physiol..

[73] Nyanasaigran L., Ramasamy S., Gautam A., Guleria P., Kumar V., Yaacob J.S. (2024). Methyl jasmonate elicitation improves the growth performance and biosynthesis of antioxidant metabolites in *Portulaca oleracea* through ROS modulation. Ind. Crops Prod..

[74] Kikowska M., Kedziora J., Krawczyk A., Thiem B. (2015). Methyl jasmonate, yeast extract and sucrose stimulate phenolic acids accumulation in *Eryngium planum* L. shoot cultures. Acta Biochim. Pol..

[75] Skrzypczak-Pietraszek E., Słota J., Pietraszek J. (2014). The influence of l-phenylalanine, methyl jasmonate and sucrose concentration on the accumulation of phenolic acids in *Exacum affine* Balf. f. ex Regel shoot culture. Acta Biochim. Pol..

[76] Dowom S.A., Abrishamchi P., Radjabian T., Salami S.A. (2017). Enhanced phenolic acids production in regenerated shoot cultures of *Salvia virgata* Jacq. after elicitation with Ag^+^ ions, methyl jasmonate and yeast extract. Ind. Crop. Prod..

[77] Abedi M., Karimi F., Saboora A. (2024). In vitro shoot multiplication of *Haplophyllum virgatum* and flavonoid elicitation in proliferated shoots by methyl jasmonate. Plant Cell Tissue Organ Cult..

[78] Zeng T., Li J., Li J., Hu H., Zhu L., Liu K., Bai J., Jiang Q., Wang C. (2024). Pyrethrins in *Tanacetum cinerariifolium*: Biosynthesis, regulation, and agricultural application. Ornam. Plant Res..

[79] Zeng T., Li J.W., Xu Z.Z., Zhou L., Li J.J., Yu Q., Luo J., Chan Z.L., Jongsma M.A., Hu H. (2022). *TcMYC2* regulates pyrethrin biosynthesis in *Tanacetum cinerariifolium*. Hortic. Res..

[80] Jeyasri R., Muthuramalingam P., Karthick K., Shin H., Choi S.H., Ramesh M. (2023). Methyl Jasmonate and Salicylic Acid as Powerful Elicitors for Enhancing the Production of Secondary Metabolites in Medicinal Plants: An Updated Review. Plant Cell Tissue Organ Cult..

[81] Antognoni F., Faudale M., Poli F., Biondi S. (2009). Methyl Jasmonate Differentially Affects Tocopherol Content and Tyrosine Amino Transferase Activity in Cultured Cells of *Amaranthus caudatus* and *Chenopodium quinoa*. Plant Biol..

[82] Beleggia R., Giovanniello V., Menga V., Suriano S., Trono D. (2026). Exogenous Application of Methyl Jasmonate Affects the Phytochemical Accumulation and the Antioxidant Activity in Hemp (*Cannabis sativa* L.) Inflorescences. Agronomy.

[83] Joshi N., Agarwal K., Ghosh S. (2023). Improved antioxidant metabolism in shoot cultures of *Ruta graveolens* (L.) in response to methyl jasmonate and abscisic acid. Plant Cell Tissue Organ Cult. PCTOC.

[84] Liu Z., Mohsin A., Wang Z., Zhu X., Zhuang Y., Cao L., Guo M., Yin Z. (2021). Enhanced biosynthesis of chlorogenic acid and its derivatives in methyl-jasmonate-treated *Gardenia jasminoides* cells: A study on metabolic and transcriptional responses of cells. Front. Bioeng. Biotechnol..

[85] Ghimire B.K., Thiruvengadam M., Chung I.M. (2019). Identification of elicitors enhances the polyphenolic compounds and pharmacological potential in hairy root cultures of *Aster scaber*. S. Afr. J. Bot..

[86] Amani S., Mohebodini M., Khademvatan S., Jafari M. (2020). *Agrobacterium rhizogenes* mediated transformation of *Ficus carica* L. for the efficient production of secondary metabolites. J. Sci. Food Agric..

[87] Fraisse D., Felgines C., Texier O., Lamaison J.L. (2011). Caffeoyl derivatives: Major antioxidant compounds of some wild herbs of the *Asteraceae* family. Food Nutr. Sci..

[88] Ganzera M., Egger C., Zidorn C., Stuppner H. (2008). Quantitative analysis of flavonoids and phenolic acids in *Arnica montana* L. by micellar electrokinetic capillary chromatography. Anal. Chim. Acta.

[89] Clauser M., Aiello N., Scartezzini F., Innocenti G., Dall’Acqua S. (2014). Differences in the chemical composition of *Arnica montana* flowers from wild populations of north Italy. Nat. Prod. Commun..

